# Genotypes of informative loci from 1000 Genomes data allude evolution and mixing of human populations

**DOI:** 10.1038/s41598-021-97129-2

**Published:** 2021-09-07

**Authors:** Sridevi Padakanti, Khong-Loon Tiong, Yan-Bin Chen, Chen-Hsiang Yeang

**Affiliations:** Institute of Statistical Science, Academia Sinica, 128 Academia Road, Section 2, Taipei, Taiwan

**Keywords:** Genome informatics, Population genetics, Computational biology and bioinformatics, Genetics

## Abstract

Principal Component Analysis (PCA) projects high-dimensional genotype data into a few components that discern populations. Ancestry Informative Markers (AIMs) are a small subset of SNPs capable of distinguishing populations. We integrate these two approaches by proposing an algorithm to identify necessary informative loci whose removal from the data deteriorates the PCA structure. Unlike classical AIMs, necessary informative loci densely cover the genome, hence can illuminate the evolution and mixing history of populations. We conduct a comprehensive analysis to the genotype data of the 1000 Genomes Project using necessary informative loci. Projections along the top seven principal components demarcate populations at distinct geographic levels. Millions of necessary informative loci along each PC are identified. Population identities along each PC are approximately determined by weighted sums of minor (or major) alleles over the informative loci. Variations of allele frequencies are aligned with the history and direction of population evolution. The population distribution of projections along the top three PCs is recapitulated by a simple demographic model based on several waves of founder population separation and mixing. Informative loci possess locational concentration in the genome and functional enrichment. Genes at two hot spots encompassing dense PC 7 informative loci exhibit differential expressions among European populations. The mosaic of local ancestry in the genome of a mixed descendant from multiple populations can be inferred from partial PCA projections of informative loci. Finally, informative loci derived from the 1000 Genomes data well predict the projections of an independent genotype data of South Asians. These results demonstrate the utility and relevance of informative loci to investigate human evolution.

## Introduction

Principal Component Analysis (PCA) is a simple yet powerful method to unravel the population structure from DNA sequences in humans or other species^1,2^. It projects the high-dimensional genotype data of individuals onto the low-dimensional space spanned by leading eigenvectors of the covariance matrix. A large number of population genetics studies indicate variations along the top principal components are strongly aligned with the differences in ethnicity, geographic locations, or environmental conditions of the subjects (e.g., Refs.^3–5^). PCA projections have therefore become a standard tool to analyze the genotype data collected from multiple populations.

Despite its utility, the primary limitation of PCA is the difficult interpretation in terms of individual Single Nucleotide Polymorphisms (SNPs). PCA is derived from the genotype data of all SNPs. It is not straightforward to pinpoint a subset of SNPs responsible for the variations along the principal components. Diametric to PCA, a rich volume of literature can be found in identifying the ancestry informative markers (AIMs) that distinguish specified populations (e.g., Refs.^6–10^). In contrast to PCA where population differences in their projections are distributed in millions of SNPs, AIM studies typically identify a small number of SNPs sufficient to delineate these populations with high accuracy. This apparent paradox is due to strong correlations of many SNPs undergoing linkage disequilibrium (LD). SNPs in the same or proximal LD blocks share identical or very similar allele frequency distributions hence can be pruned without deteriorating their power to discern populations. Some prior studies reconcile this paradox by identifying AIMs based on PCA, such as sparse PCA^11^ and PCAIMs^12^. The former imposes sparsity constraints on the contributions of SNPs to each principal component and obtains approximated PCs spanned by a few AIMs. The latter calculates the weights of SNPs to principal components in terms of their loadings (coefficients) in the corresponding Singular Value Decomposition (SVD), and randomly samples a few AIMs with probabilities proportional to the weights. Although these approaches successfully incorporate PCA information to identify AIMs, they are still aimed to find a few markers sufficient to approximate the PCA structure of the complete genotype data.

In this study, we propose an alternative criterion to identify informative loci based on PCA projections. Prior approaches all attempt to find the *sufficient* informative loci which together approximate the PCA structure of the complete data. Instead, we attempt to find the *necessary* informative loci whose removal from the data will deteriorate the PCA structure. Necessary informative loci are typically much more abundant than sufficient informative loci and densely cover the genome. These two types of informative loci serve different purposes. Sufficient informative loci are efficient in predicting population identities of subjects as they can be determined by a small panel of markers without requiring dense genotype data. By contrast, necessary informative loci accommodate rich evidence about evolution and mixing of the whole or parts of the genome. For instance, by examining the genotype distributions among the necessary informative loci we can infer whether the European and East Asian populations arise from the Eurasian common ancestors in parallel (European $\leftarrow$ Eurasian $\to$ East Asian) or in sequence (Eurasian $\to$ European $\to$ East Asian or Eurasian $\to$ East Asian $\to$ European). We can also infer the local ancestry of segments in the genomes of mixed descendants from multiple populations by comparing the necessary informative loci genotypes from reference and mixed populations. Strongly correlated SNPs under linkage disequilibrium carry redundant information about population delineations and thus are pruned from the sufficient informative loci. Yet they reveal the recombination history of individuals or populations and thus are included in the necessary informative loci.

Based on the notion of necessary informative loci, we develop algorithms to capture several aspects of evolution and mixing of human populations and conduct a comprehensive analysis of the 1000 Genomes data^13^. Projections along the top seven principal components demarcate populations at not only super-continental and continental levels (in agreement with many prior studies such as Refs.^14,15^) but also at sub-continental levels within East Asia, Africa, and Europe. Consequently, we identify the informative loci along PCs 1–7. We then approximate projections along each PC by weighted sums of genotype values over the informative loci. Furthermore, the informative loci allude various aspects about human evolution. The distributions of homozygote major/minor alleles of the informative loci along each PC reveal the directions of genotype changes during population evolution. Simulations from a simple demographic model based on several waves of founder population separation and mixing recapitulate the population distribution of projections along the top three PCs. Informative loci are distributed over the whole genome but excessively concentrate on selected hot spots, and their genes are significantly enriched in a number of functional categories. Genes located at two hot spots along PC 7 exhibit differential expressions among European populations. We also develop an algorithm to infer the local ancestry identities of tracts in the phased genotype data of a mixed subject according to the partial projections of subsets of consecutive informative loci on the genome. The tract inference outcomes of our algorithm on five mixed American populations better match the known colonization history of America and prior genotype studies than those derived from RFMix, a standard tool for local ancestry inference. Finally, we project the concatenation of the 1000 Genomes data and an external data of 168 South Asian subjects by using both PCA of the joint data and the coefficients derived from the 1000 Genomes data alone. These two approaches yield highly correlated projections, which affirms the transferability of informative markers in determining projections.

Each component in this work has been covered by similar algorithms or theoretical analysis in prior studies. PCA projections are a standard tool in population genetics. Abundant methods have been proposed to identify ancestry informative markers from genotype data, and some of them find AIMs using the information of PCA projections (see the aforementioned overview). The notion that population identities are quantitative traits which are additively determined by the sequences on many informative loci can be seen directly from the non-sparse distribution of the corresponding SVD loadings. The evolution and mixing histories of most populations covered in the 1000 Genomes Project are well characterized by decades of research utilizing rich evidence from contemporary and ancient DNAs, linguistics and archaeology. Numerous local ancestry inference algorithms are available, such as ADMIXTURE^16^, RFMix^17^, and Yang et al.^18^. Some of these algorithms incorporate information from PCA or SVD analogous to our method (e.g., Refs.^12,19,20^). Nevertheless, our work has two major contributions. First, we introduce the notion of necessary informative loci and indicate their importance in understanding the evolution and mixing history of human populations. Second, we demonstrate the utility of necessary informative loci by implementing a comprehensive analysis at a wide scope including all the populations in the 1000 Genomes data and eight aspects of their evolutionary and mixing history.

## Results

### Leading principal components of the 1000 Genomes data delineate major populations

**Figure 1:**
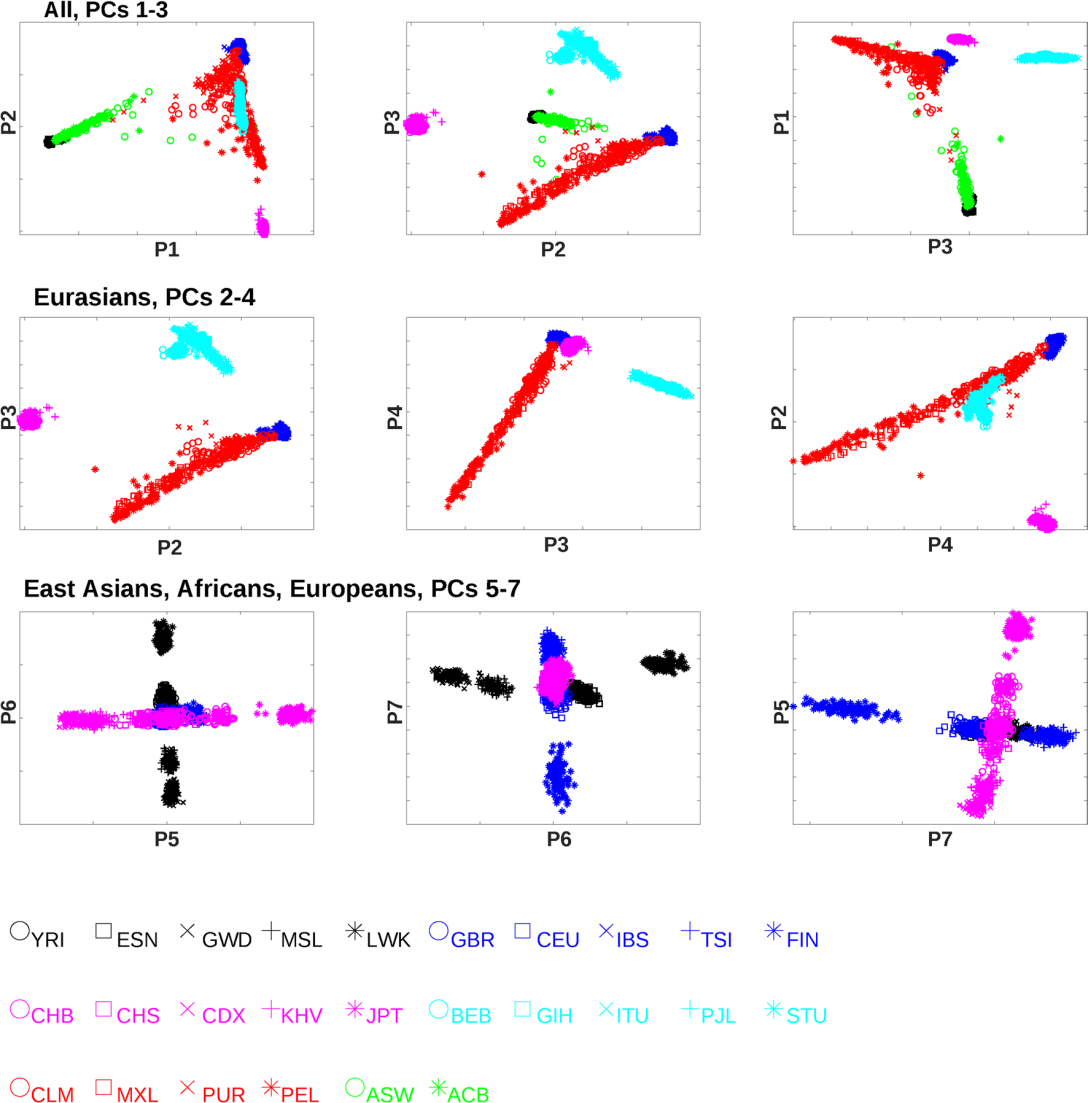
Projections of 1000 Genomes subjects along the top 7 PCs. Two-dimensional views. The top row shows all populations along PCs 1–3. The middle row shows Eurasian and Latin American populations along PCs 2–4. The bottom row shows East Asian, African and European populations along PCs 5–7 respectively. Figure generated by Matlab version 8.3.0.532 (www.mathworks.com).

We download the variant calling files (VCF) from the phase 3 data of the 1000 Genomes Project. The data comprises 2504 subjects from 26 populations across four continents. Table 1 reports the summary information of 1000 Genomes populations. They include peoples from Africa (AFR), Europe (EUR), East Asia (EAS), South Asia (SAS), and mixed populations in the American continents. We treat Peruvians (PEL) as native Americans (AMR) since their PCA projections are the furthest from Europeans and hence the closest to native Americans (Fig. 1 and Supplementary Fig. S1). The resulting data is a $30761503 \times 2504$ matrix with phased, biallelic entries ($0\left|0, 0\right|1, 1|0, 1|1$), where $0$ and $1$ denote major and minor alleles respectively.

**Table 1:** Summary of populations in 1000 Genomes data.

Population	Abbreviation	Size	Population	Abbreviation	Size
Yoruba in Nigeria	YRI	108	Kinh in Vietnam	KHV	99
Esan in Nigeria	ESN	99	Japanese in Tokyo	JPT	104
Gambian	GWD	113	African American	ASW	61
Mende in Sierra Leone	MSL	85	Afro-Carribean	ACB	96
Luhya in Kenya	LWK	99	Colombian	CLM	94
British	GBR	91	Mexican in LA	MXL	64
Utah European	CEU	99	Puerto Rican	PUR	104
Iberian	IBS	107	Peruvian	PEL	85
Toscan	TSI	107	Bengali	BEB	86
Finnish	FIN	99	Gujarati in Houston	GIH	103
Chinese in Beijing	CHB	103	Telugu in UK	ITU	102
Southern Chinese	CHS	105	Pakistani Punjabi	PJL	96
Chinese Dai	CDX	93	Tamil in UK	STU	102

We collapse the phased data by counting the number of minor alleles for each entry and construct the genotype matrix ${\varvec{X}}$ with trinary values $\{\mathrm{0,1},2\}$. ${\varvec{X}}$ is normalized by subtracting the mean of each row vector $\stackrel{\sim }{{\varvec{X}}}\equiv {\varvec{X}}-\overline{{\varvec{X}} }$. Denote $n$ and $m$ the numbers of rows (loci) and columns (subjects) in ${\varvec{X}}$, and ${\varvec{C}}\equiv {\stackrel{\sim }{{\varvec{X}}}}^{T} \cdot \stackrel{\sim }{{\varvec{X}}}$ an $m\times m$ covariance matrix of column vectors in ${\varvec{X}}$. Eigen decomposition of ${\varvec{C}}$ can be expressed as a matrix equation:

1 $${\varvec{CE}} = {{\varvec{\Lambda}}}{\varvec{E}},$$

where the diagonal matrix ${\varvec{\Lambda}}$ stores eigenvalues sorted by a decreasing order and column vectors of ${\varvec{E}}$ are the corresponding eigenvectors. The projection matrix is the concatenation of eigenvectors weighted by the corresponding eigenvalues:

2 $${\varvec{P}} \equiv {{\varvec{\Lambda}}}{\varvec{E}} = {\varvec{E}}{{\varvec{\Lambda}}},$$

where the $n$-dimensional genotype data of subject $i$ (the $i$th column in $\stackrel{\sim }{{\varvec{X}}}$) is projected onto an $m$-dimensional vector (the $i$th row in ${\varvec{P}}$).

We visually examine the projections of 1000 Genomes subjects along the top ten principal components and find the top seven PCs account for separation of populations at distinct geographic levels. Figure 1 displays the 2D projections along the top seven PCs, and Supplementary Fig. S1 displays the 3D projections along the top ten PCs. PC 1 separates Africans from Eurasians. PC 2 separates East Asians, South Asians and native Americans, and Europeans. Notably, Northern Indians (PJL and BEB) are closer to Europeans than the Southern counterparts (ITU and STU), consistent with the migration history of the subcontinent^21^. PC 3 separates native Americans, Europeans and East Asians, and South Asians. PC 4 separates native Americans and all other populations. PCs 5–7 demarcate populations within East Asia, Africa and Europe respectively. PC 5 separates two ethnic groups in/near Indochina (KHV and CDX), Han Chinese (CHB and CHS), and Japanese (JPT). PC 6 separates an East African population (LWK), two Nigerian populations (YRI and ESN), and two Far West African populations (GWD and MSL). PC 7 separates Finnish (FIN), two Northern/Western European populations (GBR and CEU), and two Southern European populations (IBS and TSI). Furthermore, on PCs 1–3 subjects of mixed populations stretch along the lines connecting their ancestral populations (Africans and Europeans for ACB and ASW, native Americans and Europeans for PUR, MXL and CLM). In contrast, PCs 8–10 carry no extra information about population demarcation. Projections along PC 8 separate African populations similar to PC 6. Projections along PCs 9–10 do not separate any population.

The power of genotype data for demarcating populations depends on the coverage of subject populations and the density of SNPs. We download and process the HapMap data^22^ (1207 subjects, 11 populations, 150,425 loci) and compute their first seven PC projections (Supplementary Fig. S2). While PCs 1–2 give rise to the same population delineation as the 1000 Genomes data, PC 3 separates the African subpopulations, PC 4 separates South Asians from other populations, and other PCs do not delineate populations. Poor population delineation is expected as the HapMap data covers much fewer SNPs and populations than the 1000 Genomes data.

### Constructing the mapping between PCA projections and genotype data

#### Determination of informative loci along each principal component

How does a 30,761,503-dimensional genotype vector transform into a 7-dimensional projection vector indicative of all major population identities? To answer this question, we represent PCA in terms of Singular Value Decomposition (SVD):

3 $$\tilde{\user2{X}} = {\varvec{U}}{{\varvec{\Sigma}}}{\varvec{V}}^{T} ,$$

where ${\varvec{U}},{\varvec{\Sigma}},{\varvec{V}}$ are $n\times n, n\times r$, and $m\times r$ matrices respectively, $r$ is the rank of $\stackrel{\sim }{{\varvec{X}}}$, and ${{\varvec{U}}}^{T}{\varvec{U}}={\varvec{I}}, {{\varvec{V}}}^{T}{\varvec{V}}={\varvec{I}}$. It can be shown that ${\varvec{V}}={\varvec{E}}$, and

4 $${\varvec{P}} = \tilde{\user2{X}}^{T} {{\varvec{\Gamma}}}, \quad {{\varvec{\Gamma}}} = \tilde{\user2{X}}\user2{V}.$$

Derivation of Eq. (4) is reported in “Materials and methods”. The projection value of subject $i$ on principal component $k$ then becomes:

5 $$P_{ik} = \mathop \sum \limits_{j = 1}^{n} \gamma_{jk} \tilde{X}_{ji} ,$$

where ${\gamma }_{jk}$ quantifies the contribution of locus $j$ to principal component $k$ and is an entry in the SVD loadings ${\varvec{U}}{\varvec{\Sigma}}$. This formulation collapses the high-dimensional genotype data (${\tilde{X }}_{ji}$ for $j=1-n$) into a small number of principal components (${P}_{ik}$ for $k=1-7$).

**Figure 2:**
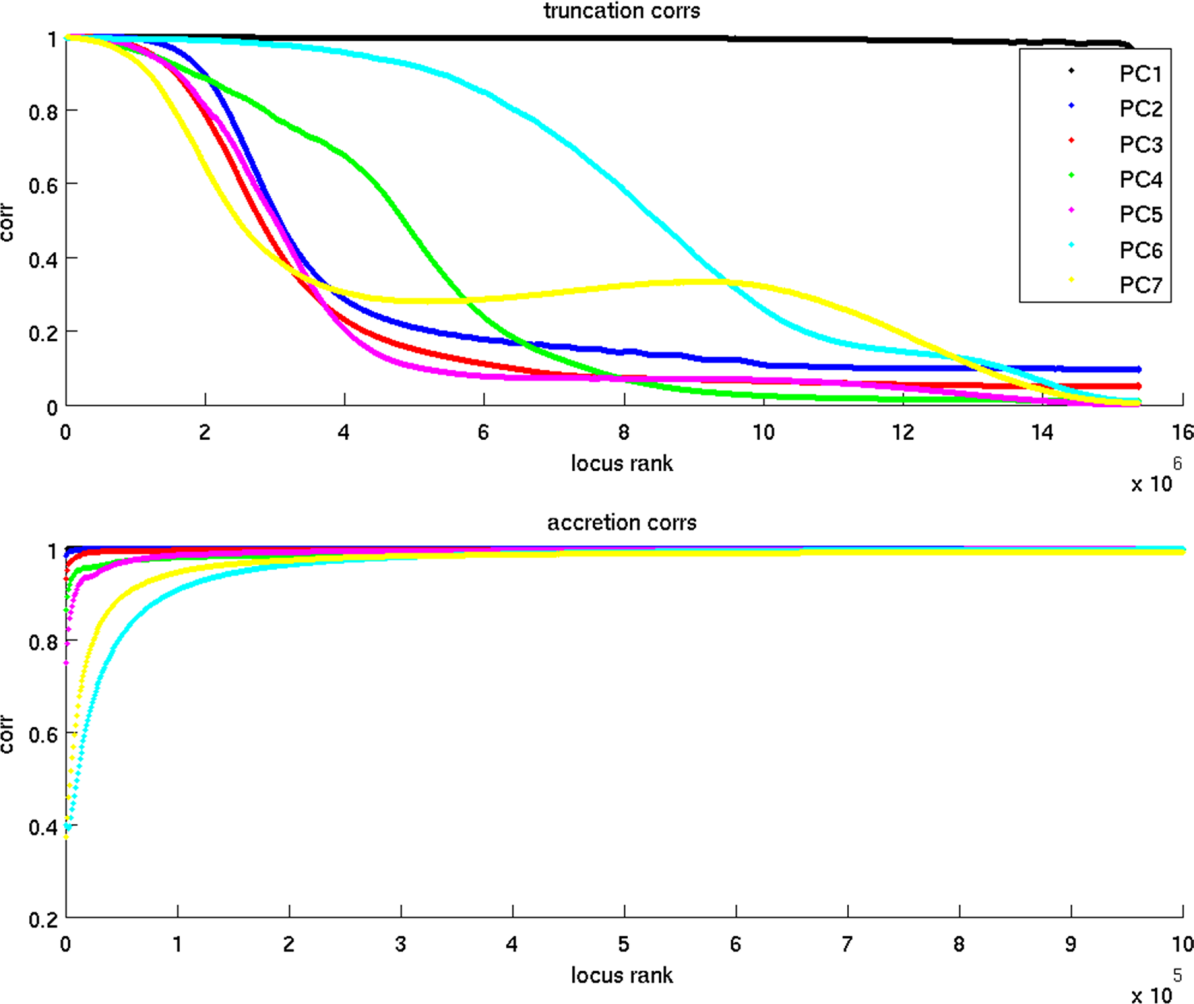
Correlation coefficients between full and approximated projection values along each PC. Top: incrementally truncate top and bottom ranking loci. Bottom: incrementally add top and bottom ranking loci. Figure generated by Matlab version 8.3.0.532 (www.mathworks.com).

The 30 million loci have unequal contributions to the principal components. In each PC, the majority of loci possess small coefficients near 0 (Supplementary Fig. S3). We suspect that only the loci with sufficiently large (positive or negative) coefficients are informative about population delineation. Those informative loci of each PC are obtained by sorting them by SVD loadings and selecting the top and bottom ones. The approximated projection values based on informative loci should resemble the projection values based on all loci. To determine the threshold for informative loci selection, we examine the correlation coefficients between the full and approximated projection values of all subjects based on two distinct selection criteria. First, we start with the full projections and incrementally remove the contributions from the top and bottom ranking loci (truncation). Second, we start with zero projection values and incrementally add the contributions from the top and bottom ranking loci (accretion). Figure 2 reports the correlation coefficients between full and two approximated projections along PCs 1–7. The truncation and accretion approaches give rise to drastically different thresholds. Correlations between full and approximated projections drop slowly with the number of truncated loci but rise rapidly with the number of accrued loci. For example, along PC 1 the full and approximated projections remain highly correlated ($r\ge 0.99$) even when $76\%$ of the top and bottom loci ($11,668,000\times 2=23,336,000$) are removed. Yet the same level of correlation is established by including only the top and bottom 1000 loci in the approximated projections. We reason that the top and bottom ranking loci selected from the truncation and accretion approaches constitute *necessary* and *sufficient* informative loci respectively. Necessary informative loci are far more abundant than sufficient informative loci because the former include all SNPs that carry non-negligible information about population differences, while the latter can be formed by a small number of positions with sufficiently strong information to delineate populations. As mentioned in “Introduction”, necessary and sufficient informative loci serve different purposes. The goals of this work are to infer the possible evolutionary and mixing history of human populations in the whole or parts of the genome. Necessary informative loci are more pertinent to fit the goals since they densely cover the genome thus have good statistical power in counting genotype differences between populations and high resolution in dissecting the genome into tracts sharing local ancestry. Therefore, we set the correlation threshold to 0.9 and select necessary informative loci for subsequent analysis. In addition, we also include the loci whose allele frequencies among the targeted populations differed by relatively large margins. The following numbers of loci along each PC are selected: PC 1: 30,714,000, PC 2: 3,958,000, PC 3: 3,132,000, PC 4: 3,614,000, PC 5: 3,212,000, PC 6: 10,674,000, PC 7: 2,380,000.

#### PCA projections are approximated by weighted sums of informative loci genotypes

Supplementary Figure S4 displays the population allele frequencies of top and bottom 10,000 informative loci along each PC. Informative loci on PCs 1–3 manifest large differences in population allele frequencies. For instance, on PC 1 the top ranking loci possess high frequencies of homozygote minor (2) and major (0) alleles in Africans and Eurasians respectively, and moderate frequencies of heterozygote alleles (1) in both populations; while the bottom ranking loci possess the opposite patterns. In contrast, on higher PCs population allele frequencies of top and bottom ranking loci demonstrate only inconspicuous differences.

How do individual loci with moderate or small population allele frequency differences collectively distinguish populations on the projections? One likely explanation is that the population identity depends on the sum of minor (or major) alleles over the informative loci. To verify this hypothesis, we construct proxy projections as weighted sums of minor/major alleles over the informative loci and compare the proxy projections of all subjects with the projections based on SVD (Eq. 5). The weight of a locus depends on the allele frequency differences between the target populations. The precise definition of the proxy weights is described in “Materials and methods”.

**Figure 3:**
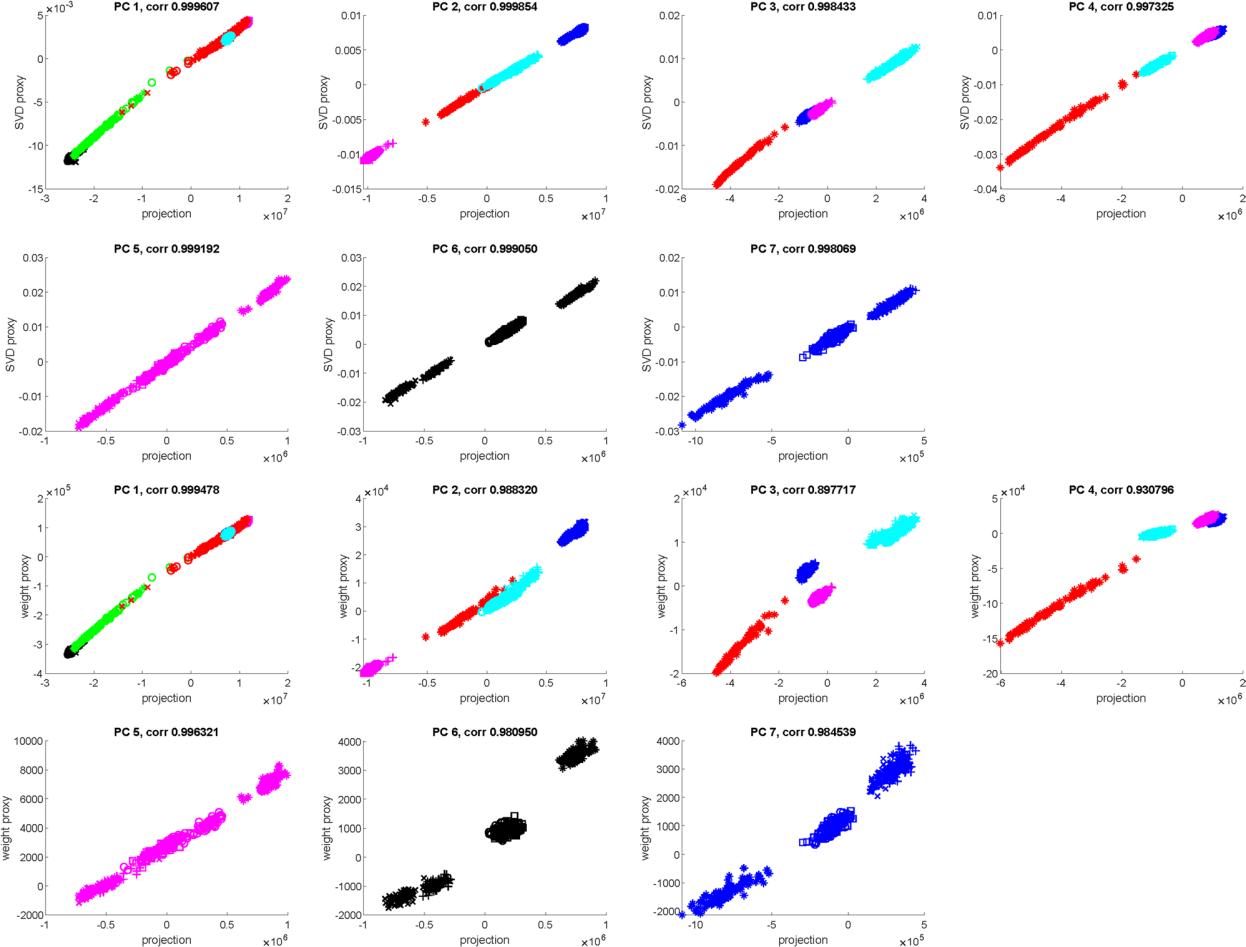
On PCs 1–7, compare the full projections using all loci (x axis) with the partial projections using SVD loadings of selected informative loci (y axis, top 7 panels), as well as the proxy projections using allele frequency weights of selected informative loci (y axis, bottom 7 panels). Figure generated by Matlab version 8.3.0.532 (www.mathworks.com).

Figure 3 displays full projections using all loci, partial projections using the SVD loadings of informative loci, and proxy projections as weighted sums of genotype values over the informative loci. Both partial and proxy projections are highly correlated with the full projections along all PCs, and hence corroborate our hypothesis.

### Variations of allele frequencies are aligned with the history and direction of population evolution

The 1000 Genomes data provide not only a snapshot of contemporary populations but also evidence about their evolution. Here we present two analyses of the 1000 Genomes data pertaining to human population evolution. First, we examine the dominant patterns of genotype changes between populations and reconstruct their phylogenetic relations. Second, we visualize the fractions of homozygote major and minor alleles among the informative loci of subjects from selected populations and impute the directions of genotype changes along each PC during population evolution.

#### Dominant patterns of genotype changes are compatible with phylogenetic relations of the 1000 Genomes populations

An alternative analysis without incurring PCA is to directly compare the population allele frequencies of all loci and detect the dominant patterns of genotype changes accordingly. For each locus, we quantize the allele frequencies of each population into a three-component binary vector. For instance, $\left(\mathrm{1,0},0\right)$ denotes that the homozygote major allele frequency considerably exceeds heterozygote and homozygote minor allele frequencies. A pattern of genotype changes is a partition of the 21 (excluding the 5 mixed populations in America) populations where each group of populations possess a unique quantized allele frequency vector. For instance, a pattern of genotype changes at super-continental level is that the African (AFR) and Eurasian (EUA) populations possess distinct quantized allele frequencies. We count the occurrences of these patterns among all loci. The algorithm of detecting the dominant patterns of genotype changes is described in Supplementary Text S1.

Supplementary Table S1 reports the sorted patterns of genotype changes of populations at super-continental, continental and subcontinental levels. Based on the occurrences of these patterns, we reconstruct the phylogenetic relations of the 21 populations and display them in Supplementary Fig. S5. The dominant patterns are compatible with the documented migration history of human populations. The quantized allele frequencies of the vast majority (more than 75%) of loci remain invariant across all populations, which manifest close relations of all human populations. Among the remaining patterns, changes between Africans (AFR) and Eurasians (EUA) far exceed changes between any other groups of populations, which support the common view that modern humans were originated from Africa and Eurasian ancestors moved out of Africa about 100,000 years ago^23,24^. Within Eurasia, two leading patterns (PEA and IEU) group East Asians (EAS) and native Americans (AMR), as well as Europeans (EUR) and South Asians (SAS) together. It is commonly accepted that ancestors of native Americans crossed the Bering land bridge from Northeast Asia during the last Ice Age^23,24^. Rich evidence from philology and archeology agrees that Indo-European ancestors moved from Central Asia east to the Indian subcontinent and west to Europe^23,24^. Within Europe, Finnish (FIN) is more distant from other Europeans, which is also supported by a smaller-scale study in Northern Europeans^25^. Northern Europeans (NEU) and Southern Europeans (SEU) are distinct partly due to the gene flow from North Africa to Southern Europe^26^. Within East Asia, Japanese (JPT) is more distinct from Han Chinese populations (CHI) and populations in/near Indochina (DKM). The closeness of Chinese ethnic minority (CDX) and Vietnamese (KHV) with Han Chinese is likely due to their frequent mixing during historical time^27^. In contrast, Japanese continuously exchanged goods and culture with mainland Asia but remained genetically isolated. Within Africa, an east African population (LWK) is distinct from other west African populations (WAF), which is also supported by a recent survey of African population genomes^28^.

#### Variations of allele frequencies on informative loci indicate the direction of population evolution

Separation of populations along certain principal components can emerge from multiple possible evolutionary trajectories. For instance, the distributions of Eurasian subject projections on PC 2 (Fig. 1, the middle left panel) can arise when (1) the common ancestors possess mostly major alleles on informative loci as in Europeans, and minor alleles gradually build up among South Asians and native Americans and eventually dominate in East Asians, (2) the common ancestors possess mostly minor alleles on informative loci as in East Asians and evolve in the opposite direction, (3) the common ancestors possess a balanced mixture of major and minor alleles on informative loci as in South Asians and native Americans, and evolve in two opposite and parallel directions toward Europeans and East Asians. We impute the directions of genotype changes along population evolution by juxtaposing the genotype distributions of the constituting populations including outgroups. Informative loci are subdivided into positive and negative groups according to the signs of their SVD loadings. We extract the high-scoring loci in both groups and count the fractions of homozygote major and minor alleles of selected loci in each group and for each subject.

**Figure 4:**
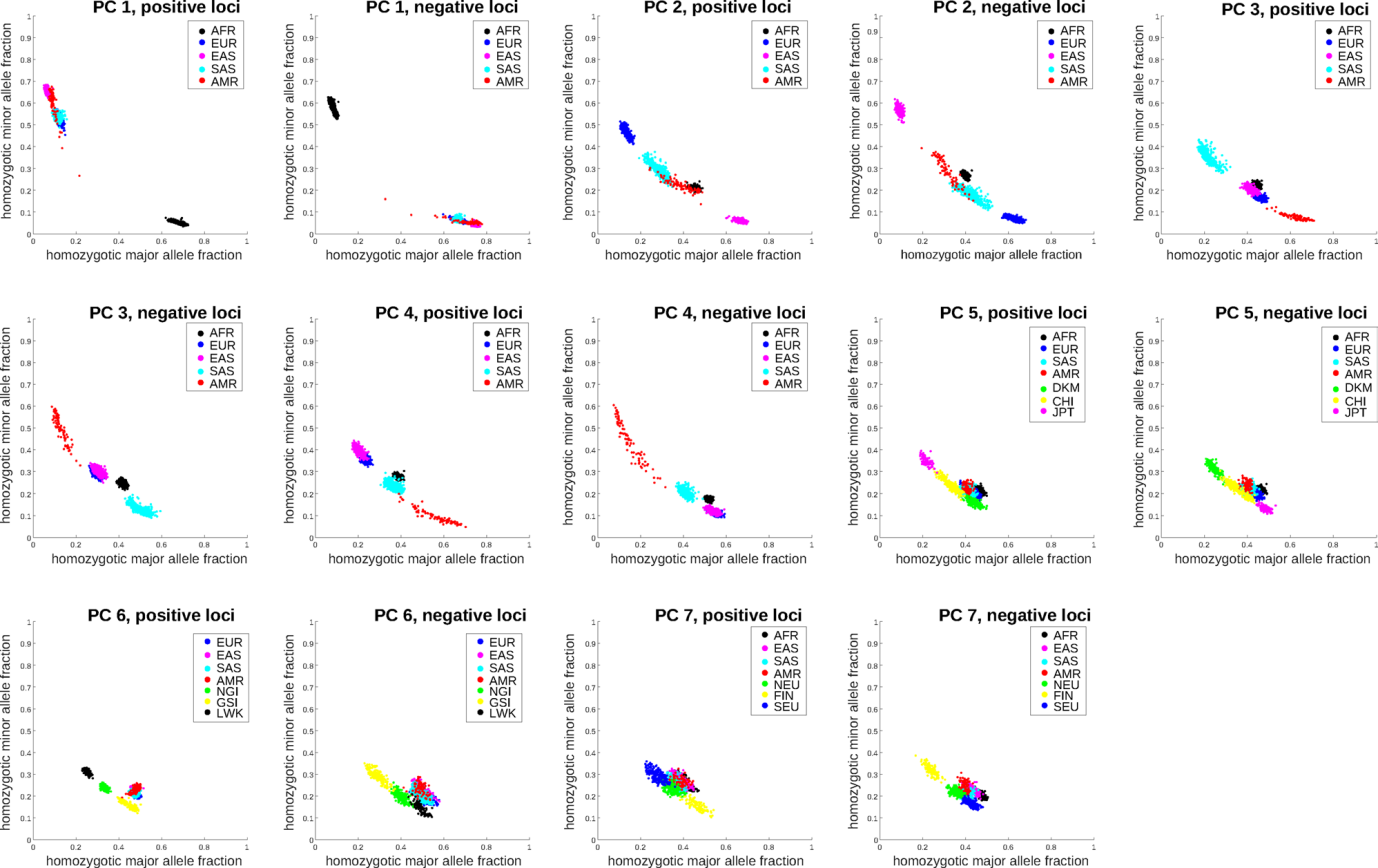
Fractions of homozygote major (x axis) and minor (y axis) alleles among the selected 200,000 informative loci and subjects from selected populations. Each dot indicates the combination of homozygote major and minor allele fractions in a subject. Each panel displays the distributions along each PC in a positive (top 100,000 informative loci) or negative (bottom 100,000 informative loci) group. Figure generated by Matlab version 8.3.0.532 (www.mathworks.com).

Figure 4 displays fractions of homozygote major and minor alleles among the top/bottom 100,000 informative loci of subjects from relevant populations along each PC. Intriguingly, evolutionary trajectories of informative loci possess diverse patterns. On both PCs 2 and 3, the outgroup subjects (Africans) lie in the middle between two extreme distributions, suggesting bidirectional evolution from a moderate mixture of genotypes in common ancestors toward two homogeneous distributions dominated by major and minor alleles. In contrast, on PCs 5 and 6, the outgroup subjects (Africans for PC 5 and Eurasians for PC 6) lie in one extreme distribution, suggesting unidirectional evolution from a homogeneous distribution dominated by major alleles in common ancestors toward another homogeneous distribution with more frequent minor alleles. On PC 7, informative loci with positive and negative SVD loadings exhibit bidirectional and unidirectional evolution respectively (the outgroups are Africans and East Asians). The evolutionary directions along PCs 1 and 4 cannot be determined since there are no outgroups.

### Simulation outcomes from a simple demographic model are compatible with the PCA projections and variations of allele frequencies on informative loci

The PCA projections and distributions of homozygote major/minor allele fractions clearly demarcate populations at super-continental, continental and subcontinental levels, yet they do not directly elucidate the evolutionary history/processes that possibly lead to the demarcation. We propose several simple demographic models based on genetic drifts and find the simulation outcomes from one model fit the PCA projections and allele frequency variations qualitatively. This simple model by no means reconstructs the evolutionary history of human populations but points out that genetic drifts plus isolation of a small founding population largely account for variations along the top three principal components.

#### Sequential and parallel models of three founding populations

We start by describing two toy models for three populations. Given the short time span pertaining to the migration and mixing of modern humans, allele frequency differences in a large number of loci between populations are most likely attributed to the genetic drifts derived from small founding populations rather than natural selection. To verify the plausibility of this hypothesis, we build simple models of population evolution based on genetic drifts and isolation of a small founding population and demonstrate that the simulated data recapitulate the patterns observed in PCA projections and homozygote allele frequency distributions. The detailed procedures of models and simulations are depicted in Supplementary Text S1. In brief, individuals with diploid genomes of 10,000 loci randomly mate within populations and reproduce progenies. A new population is formed by randomly isolating a small number of founding members from the parent population and perpetuating exponentially until reaching the capacity and maintaining a constant size onward. We consider two simplest scenarios of the formation order of three populations: (1) population 1 $\to$ population 2 $\to$ population 3 (sequential process), (2) population 2 $\leftarrow$ population 1 $\to$ population 3 (parallel process). After 20 generations we compute and visualize PCA projections of population members (as Fig. 1 for the real data) and the fractions of homozygote major and minor alleles over the top and bottom informative loci of PC 1 (as Fig. 4 for the real data).

Supplementary Figure S6 displays the projections along PCs 1 and 2 of individuals and the fractions of homozygote major and minor alleles among the top and bottom 1000 informative loci for three populations under the two models. The order of population formation is compatible with the proximity of both projection values and allele frequency distributions: 3, 2, 1 (or 1, 2, 3) for the sequential model (top panels) and 2, 1, 3 (or 3, 1, 2) for the parallel model (bottom panels). However, in the 1000 Genomes data only PC 2 agrees with the simulation outcomes of the parallel model on both PCA projections and allele frequency distributions (Figs. 1 and 4). Here populations 1, 2, 3 stand for AFR, EUR and EAS respectively. Along other PCs the analysis results from the 1000 Genomes data only partially agree with the simulation outcomes. For instance, along PC 6 the projections indicate that the out group populations (Eurasians) coincide with the NGI population in the middle, whereas the distributions of homozygote major and allele fractions indicate that the out group populations lie at one end (GSI) for positive informative loci and another end (LWK) for negative informative loci. This disparity implicates that the simple three-population model may not constitute sufficient information to account for the major sequence variations in the 1000 Genomes data.

#### A refined five-population model based on genetic drifts and population mixing

We then consider a slightly more refined demographic model that may account for the PCA projections and allele frequency distributions of the five continental-level populations (AFR, EUR, EAS, SAS, AMR). Demarcation of those populations is manifested on PCs 1–3, yet simple models based on genetic drifts of founding populations seem unable to explain the outcomes along all three principal components. While EUR and EAS are at extreme ends and SAS and AMR are in the middle along PC 2, their proximity relations are inverted along PC 3. We suspect that the founding populations of SAS and AMR come from mixtures from ancestors of EUR and EAS respectively. A demographic model of five populations is constructed accordingly (Supplementary Fig. S7). The Eurasian ancestors are first split from the AFR ancestors. Two founding populations of EUR and EAS ancestors are then split from the Eurasian ancestors. Subsequently the founding populations of SAS and AMR ancestors are formed by mixing EUR and EAS members with different proportions (60% EUR and 40% EAS for SAS, and 40% EUR and 60% EAS for AMR). Detailed procedures of model simulations are described in Materials and Methods and Supplementary Text S1. Despite its simplicity, the model is compatible with the recent findings from the genomic sequences of South Asians and indigenous Americans. South Asians are mixed descendants from two ancient populations ANI (Ancestral North Indians) and ASI (Ancestral South Indians)^21^. The former comes from West Eurasians, and the latter is unique from other populations but remotely related to East Asians. Beyond the aforementioned ancestry from Northeast Asia, indigenous Americans also inherit the gene flow from Northern European ancestors before European colonization in America^29^.

**Figure 5:**
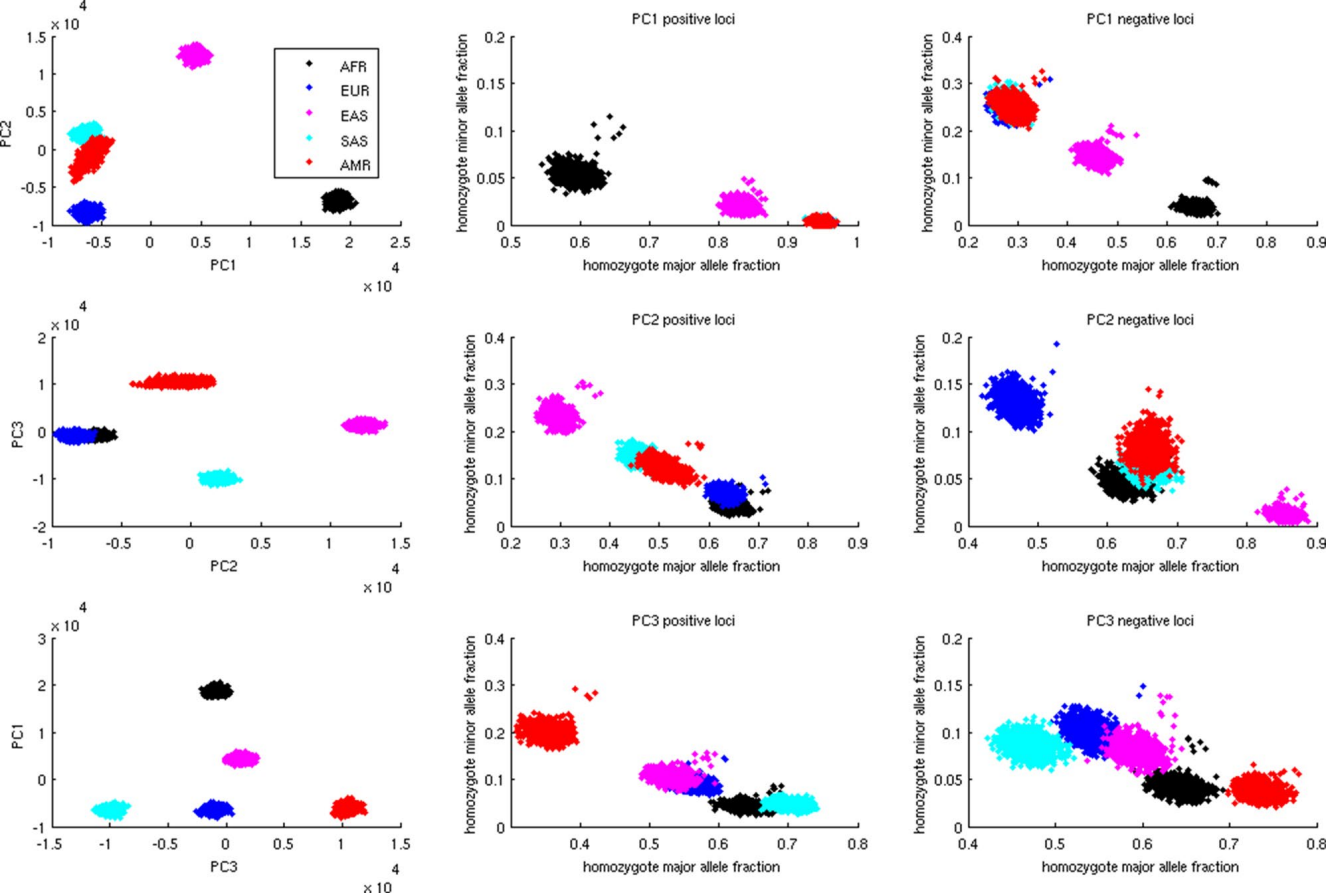
The PC projections and homozygote major and minor allele fractions of the simulation outcomes from a five-population model depicted in the text. The left panels display the two-dimensional projections of PCs 1–3. The middle panels display the homozygote major and minor allele fractions for the positive (top-ranking) loci along PCs 1–3. The right panels display the homozygote major and minor allele fractions for the negative (bottom-ranking) loci along PCs 1–3. Figure generated by Matlab version 8.3.0.532 (www.mathworks.com).

Figure 5 displays the PCs 1–3 projections and homozygote allele fractions of PCs 1–3 informative loci respectively according to simulation outcomes. They resemble those inferred from the 1000 Genomes data (Figs. 1 and 4). Along PC 1, AFR clearly separates from other populations in terms of projections and homozygote allele frequency distributions. Along PC 2, EUR and EAS are at extreme ends and other populations are in the middle for both PCA projections and homozygote allele fractions. Along PC 3, SAS and AMR are at extreme ends and other populations are in the middle for both PCA projections and homozygote allele fractions. Only the homozygote allele fractions for positive informative loci on PC 2 exhibit disparity that AFR lie at extreme ends rather than the middle.

### Informative loci possess locational concentration and functional enrichment

To further characterize positional and properties information of informative loci we perform analyses of locational concentration and functional enrichment of those loci.

#### Locational concentration of informative loci

A *hot spot* is a genomic region where the density of informative loci is significantly higher than the background density derived from all loci appeared in the 1000 Genomes VCF file. To account for position dependency of the background density, we partition each chromosome into windows of 1 Mb and elucidate the SNP densities in those windows by a Hidden Markov Model (HMM). The p-value of informative loci density in each window is calculated according to the background HMM. The detailed procedures of assessing locational concentration of informative loci are reported in “Materials and methods” and Supplementary Text S1.

Supplementary Figure S8 displays the normalized counts of informative loci at hotspots (p-values $\le {10}^{-50}$) on each PC. Hot spots of informative loci spread over many positions of all chromosomes, yet a small number of them possess excessively high levels of concentrations. Table 2 reports the prominent hot spots of informative loci concentration. The strongest concentration takes place on chr6 32–34 Mb for PCs 6 and 7. Within this 2 Mb window, the rescaled counts of informative loci are 28 and 20 times of the background rates on PC 6 and PC 7 respectively. Intriguingly, some of those hot spots harbor genes involved in immune responses, self-other identifications, and acyl-CoA metabolism. For instance, chr6 32–34 Mb encompass subunits of major histocompatibility complex, class II; chr14 106–108 Mb encompass members of immunoglobulins, chr12 112–113 Mb, chr17 43–45 Mb, and chr10 27–28 Mb encompass acyl-CoA dehydrogenase member ACAD10, acyl-CoA binding proteins ACBD4 and ACBD5 respectively. The hot spot on chr6 32–34 Mb is close to the regions with extreme African ancestry proportions in multiple Latino populations^19^.

**Table 2:** Prominent hot spots of informative loci concentration.

Location	PC	Representative genes
chr6: 32–34 Mb	5, 6, 7	HLA members
chr17: 43–45 Mb	7	ACBD4, WNT3, KIF18B
chr14: 106–108 Mb	3	IGHE, IGHG1, IGHD
chr10: 27–28 Mb	5	ACBD5
chr12: 112–113 Mb	2	ACAD10
chr11: 48–49 Mb	5	
chr6: 29–30 Mb	5	HLA-F, HLA-G, HLA-H, HLA-J
chr10: 38–40 Mb	6	ZNF33A, ZNF37A, ZNF248
chr11: 48–51 Mb	5,6	FOLH1
chr14: 48–49 Mb	4	MDGA2
chr2: 42–43 Mb	4	Cox7A2L

It is of interest to know whether genetic variations of the informative loci at the hot spots affect phenotypic variations between populations. Physiological and clinical phenotypes across populations are poorly documented. Alternatively, the Geuvadis Project^30^ provides the gene expression data of four European populations (FIN, GBR, CEU, TSI) and one African population (YRI). We treat gene expressions as molecular phenotypes and examine the phenotypic variations of three aggregate European populations (FIN, GBR + CEU as Northern Europeans or NEU, and TSI as Southern Europeans or SEU) at two hot spots pertaining to PC 7—chr6 32–34 Mb and chr17 43–45 Mb. Supplementary Table S2 reports the differential expression p-values for three population pairs at the two hot spots. 36 of 89 genes at chr6 32–34 Mb and 21 of 44 genes at chr17 43–45 Mb are differentially expressed (p-value $\le 0.001$) between at least one pair of European populations. As a control, we randomly select 500 non-overlapping 2 Mb-windows harboring 20–60 genes in the genome and perform the same differential expression analysis. 90 (18%) and 29 (5.8%) windows possess identical or higher fractions of differentially expressed genes as chr6 32–34 Mb and chr17 43–45 Mb. The results suggest that these two hot spots harbor higher fractions of differentially expressed genes among European populations compared to randomly selected regions in the genome. Procedures of differential expression analysis are described in “Materials and methods”.

#### Functional enrichment of informative loci

Besides locational preferences, we also check whether the genes encompassing the informative loci on each PC are enriched in specific functional classes. We perform functional enrichment analysis of the genes harboring the informative loci. For each PC we identify the functional classes which are enriched with the genes possessing high SVD loadings. Supplementary Table S3 summarizes the gene set enrichment analysis (GSEA) outcomes. Curiously, despite diverse functional classes are enriched in informative loci along each PC, functional groups involved in neural systems are strongly enriched in all of the 7 PCs. Enrichment of the same gene set in all PCs may result from two possible scenarios. Informative loci along distinct PCs can be located in distinct or identical member genes of the gene set. Further analysis affirms the latter. We identify 283 all-informative genes possessing high SVD loadings along all 7 PCs and report their enriched gene sets in Supplementary Table S4. The top-ranking enriched gene sets include synapse (p-value $7.29\times {10}^{-20})$, neurogenesis (p-value $4.86\times {10}^{-18}$), cell projection (p-value $3.90\times {10}^{-17}$), neuron differentiation (p-value $4.81\times {10}^{-17}$), biological adhesion (p-value $5.37\times {10}^{-14}$), and transporter complex (p-value $3.98\times {10}^{-11}$). In addition, Supplementary Fig. S9 displays the population allele frequencies of representative loci on each PC for the 283 all-informative genes. Each all-informative gene possesses distinct informative markers that distinguishes target populations along PCs 1–7.

### Informative loci are validated in external datasets

The informative loci and their constituting genotypes provide the signatures of delineating populations at different levels in the 1000 Genomes data. To demonstrate the validity of those signatures beyond the data examined, we perform two analyses by generalizing the information of those markers to independent data. First, we develop an algorithm to infer the local ancestral identities in the phased genotype data of subjects from five mixed American populations according to the partial projections of the informative loci derived from twenty one reference populations. We infer the tracts of mixed American subjects in the 1000 Genomes data and find that the tract labels are compatible with both their known migration/mixing history and the results using a well-known tract inference tool RFMix. Second, we project the concatenated data of the 1000 Genomes data and an external dataset of 168 South Asian subjects by using both PCA and the coefficients derived from the 1000 Genomes data alone. These two approaches yield highly correlated projections, which affirms the transferability of informative markers in determining projections.

#### Partial projections of informative loci deconvolve the ancestral origins of mixed subjects

As previously shown, the capacity of delineating populations is distributed among the informative loci over the entire genome. In principle, the partial projection on a chromosomal segment should also demarcate the constituting populations if it covers a sufficient number of informative loci. We exploit this property and develop an algorithm to infer the tracts of distinct ancestral identities in a mixed subject. In brief, the algorithm constitutes training, test and aggregation phases. The training data contains subjects from reference populations such as Africans and Europeans, while the test data covers subjects from mixed populations such as African Americans and Latin Americans. In the training phase, it subdivides each chromosome into minimal tracts that distinguish the reference populations in the training data. In the test phase, it calculates the partial projections on tracts of the test data and reports the most likely population labels accordingly. In the aggregation phase, it combines the tracts inferred from multiple PCs. The output constitutes locations and population labels of tracts for each mixed subject. The algorithm is described in the Materials and Methods and Supplementary Text S1.

We apply the algorithm to partition informative loci and build classifiers with the training data of 21 reference populations in the 1000 Genomes data and the test data of 5 mixed populations: PUR, MXL, CLM, ACB and ASW. To simplify analysis, we assume the ancestors of the test subjects are all from America, Africa or Europe, and ignore contributions from East and South Asia. We further subdivide African and European reference populations into subcontinental populations of GSI (GWD and MSL), NGI (YRI and ESN) and LWK for Africans, and FIN, NEU (GBR and CEU) and SEU (IBS and TSI) for Europeans respectively.

We infer tracts and assign their population labels in a hierarchical fashion. Tracts inferred from PC 1 possess African or non-African labels. Non-African tracts are subdivided into native American and European tracts according to PCs 2–4 informative loci. The tract labels inferred from PCs 2–4 may not be consistent. We resolve contradictions with two alternative criteria. Criterion 1 (relaxed criterion) assigns the labels with the strongest scores, and criterion 2 (stringent criterion) reports only the tracts with consistent population labels across PCs 2–4 and leaves all the other tracts unassigned. African and European tracts are further subdivided into subpopulation tracts according to PC 6 and PC 7 informative loci respectively.

Admixture analysis of the 1000 Genomes data using standard methods of ADMIXTURE^16^ and RFMix^17^ has been performed in a prior study^13,31^. Yet only summary information of the inference results is reported. To directly compare the performance of the two methods, we apply RFMix to the 1000 Genomes data and examine the inference results of the two methods.

**Figure 6:**
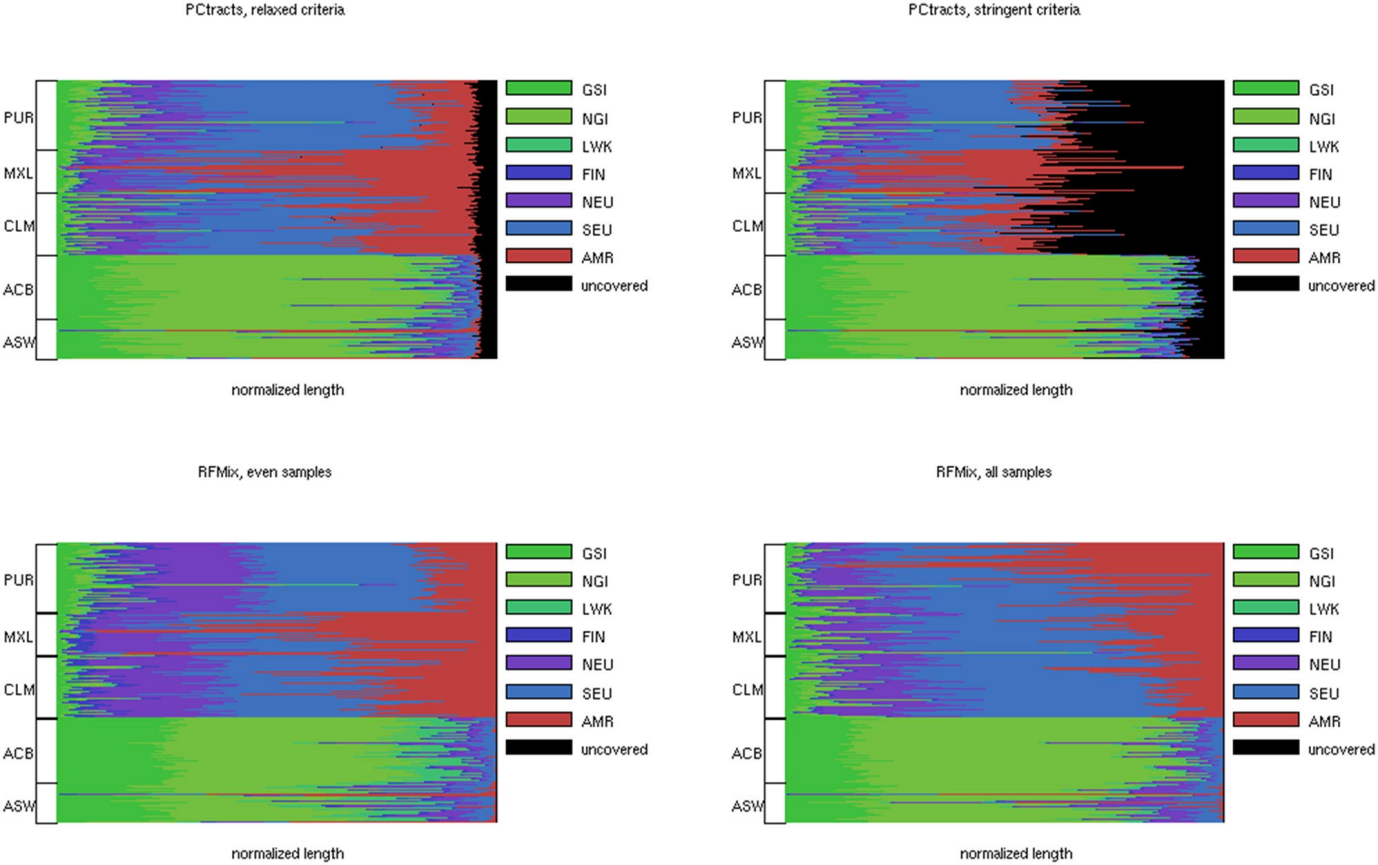
Tract length proportions from each reference population on each mixed subject using four methods of local ancestry inference. Each horizontal row displays the tract length proportions of the reference populations in a mixed subject. Black bars indicate the length proportions of the tracts without population label assignments. The mixed subjects are grouped into 5 populations. The top left and top right panels visualize the results using two alternative criteria (relaxed and stringent) to combine the inferred tracts from multiple PCs. The bottom left and bottom right panels visualize the results from RFMix by equalizing the sizes of the three continental-level reference populations and by including all subjects from the three continental-level reference populations. Figure generated by Matlab version 8.3.0.532 (www.mathworks.com).

We infer the tracts of 22 pairs of autosomes and their population labels of 419 mixed subjects and summarize the inference results in Fig. 6 and Supplementary Table S5. The inference results of RFMix are highly sensitive to the balancedness of reference population sizes. The AMR population (PEL only, 85 subjects) is far smaller than the AFR population (LWK, YRI, ESN, GWD, MSL, 504 subjects) and the EUR population (FIN, GBR, CEU, IBS, TSI, 503 subjects). The RFMix inference results using all the subjects of the reference populations considerably deviate from the local ancestries inferred from our algorithm and the summary information reported in prior studies^13,31^. To rectify this problem, we down-sample each continental-level reference population to 85 subjects and re-run RFMix to the 1000 Genomes data. The new RFMix inference results are closer to those of our algorithm and the summary information in prior studies.

Several salient properties arise from the inferred local ancestry tracts using partial PC projections. First, relaxed criterion (top left panel) yields much less unassigned tract proportions (black bars) than stringent criterion (top right panel). Second, the five mixed populations exhibit varying levels of African, European and American lineages. The two African descent populations (ASW and ACB) both have low mixture levels of Europeans. Among the three Latin American populations, MXL, CLM and PUR possess increasing mixture levels of Europeans and decreasing mixture levels of native Americans. The American lineage is negligible among ACB and ASW, while the African lineage is considerably higher among PUR than the other two Latin American populations. Third, among the two African descent populations (ACB and ASW) the Nigerian lineage (NGI) dominates the other two African subpopulations, while among the three Latin American populations the Southern European lineage dominates the other two European subpopulations. In contrast, among the African descent populations Northern and Southern European lineages have roughly equal contributions. These properties are compatible with prior studies of the 1000 Genomes data and knowledge about the population migration and mixing history in Americas.

The inferred local ancestry tracts using RFMix have considerably different distributions from those inferred from PC projections and to some extent deviant from prior knowledge about population mixing. When including all subjects in the reference populations, PUR subjects possess far more American lineage than MXL and CLM subjects. But the SEU lineage dominates the NEU and FIN lineages in the three Latin American populations. In contrast, when equalizing the reference populations, MXL, CLM and PUR possess decreasing levels of the American lineage as the inference results using PC projections. Yet the NEU lineage constitutes a similar mixed level as the SEU lineage rather than being dominated by the SEU lineage. Hence only the inference results using PC projections are fully compatible with the prior knowledge about population mixing.

#### Approximated projections derived from informative loci of the 1000 Genomes data are verified in an independent South Asian dataset

We concatenate the 1000 Genomes data and an independent South Asian dataset and demonstrate that the SVD loadings derived from the 1000 Genomes data informative loci provide a close approximation to the PCA projection of the concatenated data. We download and process the SNP data of 168 subjects of South Asian descent^32^. The external data are far less dense than the 1000 Genomes data: 11,700,630 loci appear in the original data and 8,233,400 of them appear in both datasets. We concatenate the 1000 Genomes and the external South Asian data on the intersected loci and compute the projection of each subject with two methods. First, we apply PCA to the joint data and calculate the full projections using all intersected loci. Second, we derive the SVD loadings of informative loci from the 1000 Genomes data alone, and calculate projections of the joint data using these coefficients. Detailed procedures of evaluating these two projections are reported in Materials and Methods.

Supplementary Figure S10 displays the projections of the joint data (1000 Genomes + South Asian) along the top 7 PCs using both methods. Both projections highly resemble the projections of the 1000 Genomes data alone (Fig. 1). PC 6 and PC 7 projections are swapped in the joint data due to the proximity of the 6th and 7th eigenvalues of PCA. Furthermore, for both methods the projections of the external South Asian subjects coincide with those of the South Asian subjects from the 1000 Genomes data. These observations indicate the transferability of the information from the 1000 Genomes data to an independent dataset.

To quantify generalization along each PC, we calculate the correlation coefficients between the two types of projections of the 1000 Genomes subjects and the external South Asian subjects respectively, and report them in Table 3. Among the 1000 Genomes subjects the full and proxy projections are highly correlated along each PC, as previously indicated in Fig. 3. Among the external South Asian subjects the full and proxy projections are highly correlated only along PCs 2–4. The varying levels of concordance in the external South Asian subjects are mainly due to the differential levels of their variations along the PCs. South Asian subjects have small variations along PCs 1, 5–7. Their projections on those principal components are thus noisy and poorly correlated. In contrast, PCs 2–4 accommodate reasonable levels of variations among South Asian subjects. These variations are captured by both full and proxy projections and thus strongly correlated.

**Table 3:** Correlation coefficients between the projection vectors of the two methods among the 1000 Genomes and external South Asian subjects. Notice PCs 6 and 7 are swapped between the joint and proxy projections.

PC of the joint projection	PC of the proxy projection	1000 Genomes subjects	External South Asian subjects
1	1	0.9971	0.2321
2	2	0.9943	0.9055
3	3	0.9778	0.8892
4	4	0.9601	0.7570
5	5	− 0.9276	− 0.2988
6	7	− 0.9037	− 0.4162
7	6	0.9408	0.1629

## Discussion

In this study, we introduce a new concept of necessary informative loci based on PCA projections of genotype data across populations. Necessary informative loci complement the classical ancestry informative markers (AIMs) in their construction and functions. PCA projections of the data restricted to AIMs (or sufficient informative loci by our definition) approximate those of the complete data. In contrast, PCA projections of the data removing necessary informative loci are substantially less correlated with those of the complete data. Based on the notion of necessary informative loci, we conduct a comprehensive analysis to the 1000 Genomes data and capture several aspects of evolution and mixing of human populations. Projections along the top seven principal components demarcate populations at super-continental, continental, and sub-continental levels. Necessary informative loci along each principal component comprise millions of SNPs. Projection values along each PC are approximated by weighted sums of genotype values over the informative loci, where the weights are determined by allele frequency differences between the demarcated populations. Furthermore, the informative loci elucidate various aspects of split and mixing of human populations. Allele frequency distributions of informative loci of each PC implicate the heterogeneous directions of genotype changes during the evolution of human populations. Projections and genotype distributions of five populations (AFR, EUR, EAS, SAS, AMR) along PCs 1–3 are recapitulated by simulations from a simple demographic model based on several waves of founder population separation and mixing. The informative loci are significantly concentrated in certain hot spots of the genome (such as chr6 32–34 Mb harboring HLA genes for PCs 5–7) and enriched in certain functional classes (such as neural systems for all PCs). Genotypic variations of informative loci on hot spots may also affect phenotypic variations, as genes located on two hot spots (chr6 32–34 Mb and chr17 43–45 Mb) exhibit differential expressions among three European populations. Furthermore, we develop an algorithm to deconvolve the genome of a mixed descendant from multiple populations into the tracts of distinct ancestral origins using partial projections of necessary informative loci. Finally, we validate the transferability of informative loci by demonstrating that their SVD loadings suffice to recapitulate the PC projections of an external genotype dataset of South Asian populations.

Our study of informative loci confirms an intuitive interpretation of PCA on genotype data and a common view about the origin and evolution of human populations. Population identities are an aggregate outcome derived from a large number of informative loci distributed in the entire genome. Except for populations arising from early splits of human evolution (such as Africans versus non-Africans), individual markers are unable to perfectly segregate the target populations (Supplementary Fig. S4), yet the projections based on a collection of those markers can. This property resembles the quantitative traits such as heights, psychological attributes and risks of complex diseases, which are jointly and additively determined by a large number of loci in the genome. SVD loadings of the informative loci are proportional to their contributions to population delineation. The match of observed patterns of top three PCA projections and homozygote allele fractions with simulation outcomes from a simple demographic model suggests that genotype differences among populations are likely attributed to genetic drifts and mixing from a large number of loci in small founding populations. A few individuals are isolated from single or multiple populations. The allele frequencies on many loci of these founding members are distinct from those of the parent populations by random chance. The founding members independently perpetuate and grow into another population, and the loci carrying the initial allele frequency differences become informative loci. These two views have been widely recognized by population geneticists and evolutionary biologists. For instance, McVean finds relations of coalescence time with linkage disequilibrium^33^ and PCA projections^34^. In addition, genetic drifts and geographic or ecological isolation are viewed as the fundamental processes of speciation^35^. We verify these propositions in a large genotype data of human populations (1000 Genomes data) and identify the SNPs putatively responsible for population splits during their evolution.

The distributive nature of informative loci renders them amenable to dissect the genome of a mixed descendant from multiple populations into the tracts of distinct local ancestries. Abundant local ancestry inference algorithms have been proposed (e.g., ADMIXTURE^16^, RFMix^17^), including ones that also use SVD loadings or PCA^19,20^. Our algorithm resembles PCAdmix^19^ as both methods utilize partial projections of subsets of consecutive loci to discern local ancestry. Yet there are also several fundamental differences between the two methods. PCAdmix assumes a one-to-one mapping between each principal component and each ancestral population (i.e., an ancestral population has high projection values along one PC). Our method does not hold this strong assumption and thus allows more flexible relations between populations and PCs, such as the presence of multiple populations along the gradient of one PC (e.g., FIN, NEU, SEU). The ancestral populations are multiple independent entities for PCAdmix but are organized as a hierarchy with varying levels of details for our method. Hence our analysis reports tracts of non African-European-Southern European separately. PCAdmix uses windows of a fixed size (number of SNPs), but our algorithm chooses windows with varying sizes where partial projections in each window maintain minimal separation of the ancestral populations. Despite these differences (and the differences with other local ancestry inference methods), we do not claim superiority of informative loci to the state-of-the-art methods. Rather, we intend to demonstrate that the partial information contained in the informative loci can impute missing attributes such as local ancestry of mixed subjects or PCA projections of external subjects. Nevertheless, for local ancestry inference, partial projections based on informative loci have one advantage over likelihood based methods such as RFMix, since SVD loadings and partial projections are not highly sensitive to the reference population sizes, but likelihood scores are.

Despite the remarkable fit to the patterns of genotype changes, PCA projections, and distributions of homozygote major and minor alleles, our simple models (Supplementary Figs. S5 and S7) by no means capture the true evolutionary history of human populations, which contains many subtleties hardly identifiable from the 1000 Genomes data alone. For example, separation of Northern and Southern Europeans can be partly due to differential mixing with other populations (such as mixing of Southern European and North African populations) rather than their split from the common ancestors. Likewise, proximity of Han Chinese and Vietnamese relative to Japanese can be partly due to the continuous mixing of the former two populations and relative isolation of the third, rather than early split of Japanese from mainland East Asians. In addition, although mutations and natural selection are ignored in this study, they are known to play critical roles in human evolution albeit often confined to specific genomic regions. An illuminating example concerns hot spots of informative loci. These hot spots have excessively high concentrations of informative loci, and some of them harbor genes involved in immune responses, self-other identifications, and other metabolic processes. Furthermore, genes located at chr6 32–34 Mb and chr17 43–45 Mb, two informative loci hot spots of PC 7, exhibit differential expressions among the Finnish, Northern and Southern European populations in the RNAseq data. The results implicate that genetic variations on these hot spots may affect their molecular phenotypes (gene expressions), which may undergo natural selection. To sum up, more evidence from the genomes of contemporary and ancient populations as well as linguistics, archeology, anthropology and environmental science is required in order to acquire a more complete picture of population evolution.

## Materials and methods

### 1000 Genomes data processing

We download the variant calling files (VCF) from the phase 3 data of the 1000 Genomes Project^13^. We select the loci according to the following criteria: (1) they are located on autosomes, (2) they are biallelic among the subjects, (3) they have valid entries in more than 90% of the subjects. 30,761,503 loci meet those conditions. The resulting data is a $30,761,503\times 2504$ matrix with phased, biallelic entries (0|0, 0|1, 1|0, 1|1), where 0 and 1 denote major and minor alleles respectively. We further add the biallelic entries of each locus to form a genotype matrix ${\varvec{X}}$ which take values in $\{\mathrm{0,1},2\}$. The genotype matrix is used in PCA, and the phased data is used in tract inference analysis.

### Evaluating PCA projections

We first express covariance matrix ${\varvec{C}}$ in terms of SVD decomposition:

6 $${\varvec{C}} = \tilde{\user2{X}}^{T} \tilde{\user2{X}} = {\varvec{V}}{{\varvec{\Sigma}}}^{T} {\varvec{U}}^{T} {\varvec{U}}{{\varvec{\Sigma}}}{\varvec{V}}^{T} = {\varvec{V}}{{\varvec{\Sigma}}}^{T} {{\varvec{\Sigma}}}{\varvec{V}}^{T} .$$

This is the diagonalization form of ${\varvec{C}}$. Thus ${\varvec{V}} = {\varvec{E}}$ and ${{\varvec{\Sigma}}}^{T} {{\varvec{\Sigma}}} = {{\varvec{\Lambda}}}$. The projection matrix in Eq. (2) can be re-expressed as:

7 $$\tilde{\user2{X}}^{T} {\varvec{U}}{{\varvec{\Sigma}}} = {\varvec{V}}{{\varvec{\Sigma}}}^{T} {\varvec{U}}^{T} {\varvec{U}}{{\varvec{\Sigma}}} = {\varvec{V}}{{\varvec{\Sigma}}}^{T} {{\varvec{\Sigma}}} = {\varvec{E}}{{\varvec{\Lambda}}} = {\varvec{P}}.$$

The projection vector of the $i$th subject (the $i$th row of ${\varvec{P}}$) is the linear combination of his/her normalized genotypes (the $i$th column of $\stackrel{\sim }{{\varvec{X}}}$) weighted by a loading matrix ${\varvec{\Gamma}}\equiv {\varvec{U}}{\varvec{\Sigma}}$. Direct evaluation of SVD is intractable since $n\gg m$. Nevertheless, ${\varvec{\Gamma}}$ is simply the product of the normalized genotype data and the eigenvector matrix:

8 $$\tilde{\user2{X}}\user2{V} = {\varvec{U}}{{\varvec{\Sigma}}}{\varvec{V}}^{T} {\varvec{V}} = {\varvec{U}}{{\varvec{\Sigma}}} = {{\varvec{\Gamma}}}.$$

PCA is computed by both invoking PLINK^36^ to the genotype matrix and directly evaluating the covariance matrix ${\varvec{C}}$ by our customized C program and performing eigen decomposition in Matlab. The two methods yield highly correlated results, so we report the results from our own calculation.

### Selecting informative loci

For each PC, a locus is selected as an informative locus if it satisfies one of the following conditions. First, it is among the top and bottom ranking loci such that the correlation coefficient between the full and partial projections by truncating the top/bottom ranking loci $\ge 0.9$. Second, its allele frequencies separate the target populations along the PC. Suppose along the $k$th PC there are $L$ reference populations, and the relative allele frequency of locus $i$ on population $l$ is ${f}_{kl}^{i}\equiv \left({f}_{kl0}^{i},{f}_{kl1}^{i},{f}_{kl2}^{i}\right), \sum_{g=0}^{2}{f}_{klg}^{i}=1$. The mean ${\mu }_{ik}^{l}$ and standard deviation ${\sigma }_{ik}^{l}$ according to each ${f}_{kl}^{i}$ can be calculated by a multinomial distribution. We sort the $L$ populations in terms of their projections along the PC (Fig. 1, suppose they follow the order $\mathrm{1,2},\dots L$) and check whether the the mean genotype values ${\mu }_{ik}^{1},\dots ,{\mu }_{ik}^{L}$ are either monotonically increasing or monotonically decreasing. Furthermore, for $L=2$ we also require the intervals $({\mu }_{ik}^{l}-$ ${\sigma }_{ik}^{l},{\mu }_{ik}^{l}+$ ${\sigma }_{ik}^{l})$ between adjacent populations do not overlap.

### Approximating PCA projections as weighted sums of informative loci genotypes

We approximate projections along a PC as weighted sums of genotypes over the informative loci. On the $k$th PC suppose there are $L$ reference populations (e.g., East Asians, South Asians + native Americans, and Europeans on PC2). For an informative locus $i$ denote ${f}_{klg}^{i}$ the relative frequency of allele $g$ in the reference population $l$. We compute the expected genotype value over members of the reference population:

9 $$\mu_{ik}^{l} = \mathop \sum \limits_{g = 0}^{2} f_{klg}^{i} \cdot g.$$

Intuitively, locus $i$ carries more weight in the projection if its expected genotype values of the reference populations follow the order depicted in Fig. 1 and are separated by large margins. To quantify this intuition, we first check whether the ${\mu }_{ik}^{l}$ scores follow this monotonic order (for instance, ${\mu }_{i2}^{\text{EAS}}\le {\mu }_{i2}^{\text{SAS+AMR}}\le {\mu }_{i2}^{\text{EUR}}$). If so, we then sort the reference populations by their order and define the weight of locus $i$ on the $k$th PC as the minimal margin over pairs of consecutive reference populations:

10 $$w_{ik} = sign\left( {\gamma_{ik} } \right)\mathop {\min }\limits_{{l = 1 - \left( {L - 1} \right)}} | \mu_{ik}^{l} - \mu_{ik}^{l + 1} |$$

and ${w}_{ik}=0$ if the ${\mu }_{ik}^{l}$ scores do not follow this monotonic order. The proxy projection of subject $i$ on PC $k$ is the weighted sum of his/her genotypes over the informative loci:

11 $${\widehat{P}}_{ik}=\sum_{j\in \{{\text{informative loci of }PC}_{k}\}}{w}_{jk}{\tilde{X }}_{ji}.$$

### Inferring and counting the occurrences of patterns of genotype changes

We develop an algorithm to detect the dominant patterns of genotype changes from the 1000 Genomes data. The inputs are the genotype data and population labels of all subjects. The outputs are a sorted list of dominant patterns of genotype changes and their occurrence counts. The detailed procedures of the algorithm are described in Supplementary Text S1.

### Visualizing fractions of homozygote alleles among informative loci

Along each PC, we count the fractions of homozygote major and minor alleles among the top or bottom 100,000 informative loci for each subject. The homozygote allele fractions of the subjects from selected populations are displayed on two-dimensional planes and colored according to their population labels.

### Simulating population splitting and mixing

To find the evolutionary processes that possibly explain the genotype characteristics of the 1000 Genomes data, we build simple evolutionary models of three and five populations and demonstrate that their simulation outcomes recapitulate the PCA projections and homozygote allele fraction distributions of the observed data. The primary assumptions of the models are (1) recombination is the dominant evolutionary mechanism, and sequence mutation plays a negligible role, (2) a new population is originated from a few founding members who are either drawn from one parent population or mixed from two parent populations, (3) mixing takes place only during the founding population formation, while inbreeding within populations occurs in the remaining time. The detailed descriptions of the model and simulation procedures are reported in Supplementary Text S1.

### Detecting locational concentration of informative markers

To detect the hot spots in the genome with excessive densities of informative loci, we first construct an HMM describing the location-varying background density of all SNPs. We subdivide each chromosome into windows of 1 Mb and count the number of SNPs within each window. The background model assumes that the SNP count within each window follows a Poisson distribution, and consecutive windows tend to possess similar Poisson rates. The p-value is the probability that the loci count within a window exceeds the count of informative loci given the background model. We define hotspots as windows with p-values $\le {10}^{-50}$. The detailed descriptions of the background HMM and p-value calculation are reported in Supplementary Text S1.

### Quantifying differential expressions of genes at two informative loci hot spots of PC 7

Gene expression data of five 1000 Genomes populations (95 FIN, 91 CEU, 94 GBR, 93 TSI, 89 YRI subjects) are generated from the Geuvadis Project^30^ and deposited at ArrayExpress database^37^, http://www.ebi.ac.uk/arrayexpress/, accession number E-GEUV-1). The transcript level read count data (https://www.ebi.ac.uk/arrayexpress/files/E-GEUV-1/GD660.TrQuantCount.txt.gz) is processed and p-values of three pairwise population comparisons (FIN and CEU + GBR, FIN and TSI, TSI and CEU + GBR) of differential gene expressions are calculated using DESeq2^38^. Two hot spots—chr6 32–34 Mb and chr17 43–45 Mb—contain informative loci along PC 7 separating European populations. At each hot spot we count the fraction of differentially expressed genes (DEG, p-value $\le 0.001$). To assess the significance of enrichment with differentially expressed genes, we randomly select 500 non-overlapping 2 Mb-windows containing 20–60 expressed genes, count their fractions of differentially expressed genes, and count the numbers of the control windows possessing higher fractions of differentially expressed genes than the two hot spots.

### Assessing functional enrichment of informative markers

Standard functional enrichment analysis such as hypergeometric tests or GSEA operate at gene level: the basic elements are genes. The genotype data, instead, are locus based: the basic elements are loci, and one gene may possess multiple loci. There is a freedom to adopt gene based or locus based functional enrichment analysis. The former aggregates the scores of multiple loci in the same gene into one score. The latter treats all loci of the genes in the same functional class as its unique members. We adopt a gene based approach since it dilutes the inflated importance of genes harboring multiple informative loci.

We first assess functional enrichment for informative loci along each PC. All genes harboring the informative loci are selected. Here we define the score of a gene as the maximum over the absolute values of the SVD loadings in the constituting loci. Standard GSEA is applied to the informative loci on each PC and each gene set from the MSigDB database^39^. Both the test statistics (the maximum gap between the random walks of the empirical data and a null model) and the Kolmogorov–Smirnov (KS) p-values of enriched gene sets (p-values $\le {10}^{-10}$ along at least one PC) are reported in Supplementary Table S3.

We then identify the genes that harbor informative loci along all seven PCs and assess functional enrichment of those all-informative genes. The score of a gene along a PC is previously defined. We sort genes by their scores along each PC and define a gene as all-informative if its score along each PC is in the top 25% of the sorted gene list. 283 all-informative genes are identified accordingly. We perform Fisher’s exact test on those 283 genes and report the top-ranking gene sets in terms of their hyper-geometric p-values in Supplementary Table S4.

### Deconvolving tracts of mixed subjects based on partial projections of informative markers

The capacity of delineating populations is distributed among the informative loci over the entire genome. In principle, the partial projection on a chromosomal segment should also demarcate the constituting populations if it covers a sufficient number of informative loci. We exploit this property and develop an algorithm to infer the tracts of distinct ancestral identities in a mixed subject. In brief, the algorithm constitutes training, test and aggregation phases. The training data contains subjects from reference populations such as Africans and Europeans, while the test data covers subjects from mixed populations such as African Americans and Latin Americans. In the training phase, it subdivides each chromosome into minimal tracts that distinguish the reference populations in the training data. In the test phase, it calculates the partial projections on tracts of the test data and reports the most likely population labels accordingly. In the aggregation phase, it combines the tracts inferred from multiple PCs. The output constitutes locations and population labels of tracts for each mixed subject. Detailed procedures of the algorithm are reported in Supplementary Text S1.

### Approximating the projections of an independent South Asian dataset with the coefficients derived from the 1000 Genomes data alone

To verify the utility of the informative loci beyond the 1000 Genomes data, we concatenate the 1000 Genomes data and an independent South Asian dataset and demonstrate that the SVD loadings derived from the 1000 Genomes data informative loci provide a close approximation to the PCA projection of the concatenated data.

#### Processing of the independent South Asian data

We download an independent genotype dataset of 168 South Asian subjects reported in Chambers et al.^32^. The VCF file of 11,700,630 loci is provided in the website https://www.ebi.ac.uk/ena/data/view/PRJEB57368. We consider the intersection of the loci in the VCF files of the 1000 Genomes and the external South Asian data and filter out the loci which have different definitions of major and minor allele sequences between the two VCF files. 8,233,400 loci are selected and the joint data of 2504 1000 Genomes subjects and 168 external South Asian subjects are generated accordingly.

#### Computations of the PCA projections of the joint data

We compute the PCA projections of the joint data using two methods. Method 1 employ eigen decomposition to the covariance matrix of the normalized concatenated data (Eq. 1), and construct the projection matrix as the concatenation of eigenvectors weighted by the corresponding eigenvalues (Eq. 2). Method 2 obtains the projection coefficients of informative loci from the 1000 Genomes data alone, and uses them to evaluate the projection values of the joint data. To be precise, denote $\stackrel{\sim }{{\varvec{X}}}$ the normalized 1000 Genomes data, ${\varvec{V}}$ the concatenation of eigenvectors of its covariance matrix, and ${\varvec{\Gamma}}=\stackrel{\sim }{{\varvec{X}}}{\varvec{V}}$. Furthermore, denote ${\stackrel{\sim }{{\varvec{X}}}}^{J}$ the normalized joint data, ${\gamma }_{jk}$ the SVD loadings derived from the 1000 Genomes data (Eq. 4), and ${\mathcal{I}}_{k}$ the set of informative loci along the $k$th PC. The projection value of subject $i$ of ${\stackrel{\sim }{{\varvec{X}}}}^{J}$ on principal component $k$ is approximated by

12 $${\tilde{P }}_{ik}^{J}=\sum_{j\in {\mathcal{I}}_{k}}{\gamma }_{jk}{\tilde{X }}_{ji}^{J}.$$

### Data analysis programs

The source codes of four data analysis programs and example data of running those programs are deposited under synapse.org/#!Synapse:syn25946110/files/Yeang_1000G_programs.zip.

## Supplementary Information

Supplementary Information.
